# Automated proper lumping for simplification of linear physiologically based pharmacokinetic systems

**DOI:** 10.1007/s10928-019-09644-5

**Published:** 2019-06-21

**Authors:** Shan Pan, Stephen B. Duffull

**Affiliations:** 1 School of Pharmacy, University of Otago, Dunedin, New Zealand; 2 St John’s Institute of Dermatology, Guy’s and St Thomas’ NHS Foundation Trust, Great Maze Pond, London, SE1 7EH UK

**Keywords:** Model simplification, Proper lumping, Autolumping, Physiologically based pharmacokinetic models, Systems models

## Abstract

Physiologically based pharmacokinetic (PBPK) models are an important type of systems model used commonly in drug development before commencement of first-in-human studies. Due to structural complexity, these models are not easily utilised for future data-driven population pharmacokinetic (PK) analyses that require simpler models. In the current study we aimed to explore and automate methods of simplifying PBPK models using a proper lumping technique. A linear 17-state PBPK model for fentanyl was identified from the literature. Four methods were developed to search the optimal lumped model, including full enumeration (the reference method), non-adaptive random search (NARS), scree plot plus NARS, and simulated annealing (SA). For exploratory purposes, it was required that the total area under the fentanyl arterial concentration–time curve (AUC) between the lumped and original models differ by 0.002% at maximum. In full enumeration, a 4-state lumped model satisfying the exploratory criterion was found. In NARS, a lumped model with the same number of lumped states was found, requiring a large number of random samples. The scree plot provided a starting lumped model to NARS and the search completed within a short time. In SA, a 4-state lumped model was consistently delivered. In simplify an existing linear fentanyl PBPK model, SA was found to be robust and the most efficient and may be suitable for general application to other larger-scale linear systems. Ultimately, simplified PBPK systems with fundamental mechanisms may be readily used for data-driven PK analyses.

**Electronic supplementary material:**

The online version of this article (10.1007/s10928-019-09644-5) contains supplementary material, which is available to authorized users.

## Introduction

The human body is complex by nature with hierarchical levels in structural organisation, ranging from atom to molecule, cell, tissue, organ, system, and up to the highest whole body level (refer to [1] for more details). Understanding the human body structures, more specifically biological, physiological, pathophysiological and pharmacological structures, enables the development and emergence of systems models. Two separate types of systems models, physiologically-based pharmacokinetic (PBPK) models and systems pharmacology models, may be used to quantify the interactions of drugs within the human body. These models are highly complicated, often nonlinear and evolve consistently with growing knowledge of the human systems. Contributions of these models have been recognised, for example to drug development from the early drug discovery stage to the later clinical trial stage [2, 3].

Physiologically-based pharmacokinetic (PBPK) models describe the drug pharmacokinetics (PK) in physiological systems, using a large number of compartments representing tissues and organs that are realistic to physiological systems [4]. PBPK models have been widely used in pre-clinical settings in order to predict the PK behaviour of an investigational drug in humans before commencement of first-in-human studies. The use of PBPK models for extrapolation to special populations, e.g. paediatrics and the obese, is also commonly seen [5].

Due to high dimensionality and structural complexity, systems models are not readily utilised for data-driven population PK or PKPD studies that are routinely based on empirical estimation-based approaches. With model order reduction methods, however, these models can be reduced into fewer states and simpler structures while maintaining fundamental mechanisms and important input–output relationships [6]. The simpler structures are easier to manipulate and are amenable to data-driven estimation-based techniques.

Different model order reduction techniques, including time-scale analysis, sensitivity analysis and lumping, have been proposed and applied in the literature [6]. The first two techniques may alter the kinetics of an original system, via ad hoc analysis for manual separation of time scales or elimination of states. Lumping is a potentially more flexible technique for linear or linearised models where the merging of states is determined in relation to the performance characteristics of the model. Lumping was used to simplify a PBPK model for barbiturates in rats where tissues with identical structural specification (i.e. in serial or parallel connection) and similar kinetics were merged [7]. Similarly, mathematical transformation was also adopted by Pilari and Huisinga [8] to manually simplify perfusion and permeability rate limited PBPK models, and the descriptive performance of simplified models using the lumping approach were further evaluated with 25 diverse small molecules.

Proper lumping is a special case of lumping that merges some original states into only one pseudo-state in a reduced system. The reduced states, after proper lumping, maintain their physical meaning as in the original system, as do the associated parameters. Recently, a proper lumping technique has been used for the simplification of a large-scale systems pharmacological model for a coagulation network and [9, 10].

Simplification of systems models using proper lumping technique may be automated, but represent a large-scale combinatorial search problem. An intuitive and straightforward approach is to conduct an exhaustive search over the entire solution space. While, in theory guaranteed to locate the best solution, an exhaustive search is generally not practical due to computational limitations [11], and efficient search algorithms that are scalable to large combinatorial problems are therefore desirable. Monte Carlo sampling method requires random samples repeatedly drawn to find the best solution and a greater number of random samples is expected to provide a greater probability of success [12]. Simulated annealing is a probabilistic algorithm that converges asymptotically to the optimal solution and is robust for both continuous and discrete search problems [13], and continuous search methods using SA, for example, have been used for optimising sampling times in PKPD experiments [14].

The current study aimed to explore automated proper lumping methods for the simplification of large systems models, by applying to an existing linear PBPK model for fentanyl. Here fentanyl is being used as an example and this work is not intended to form the basis of further analyses of fentanyl pharmacokinetics.

## Materials and methods

### Fentanyl PBPK model

**Fig. 1:**
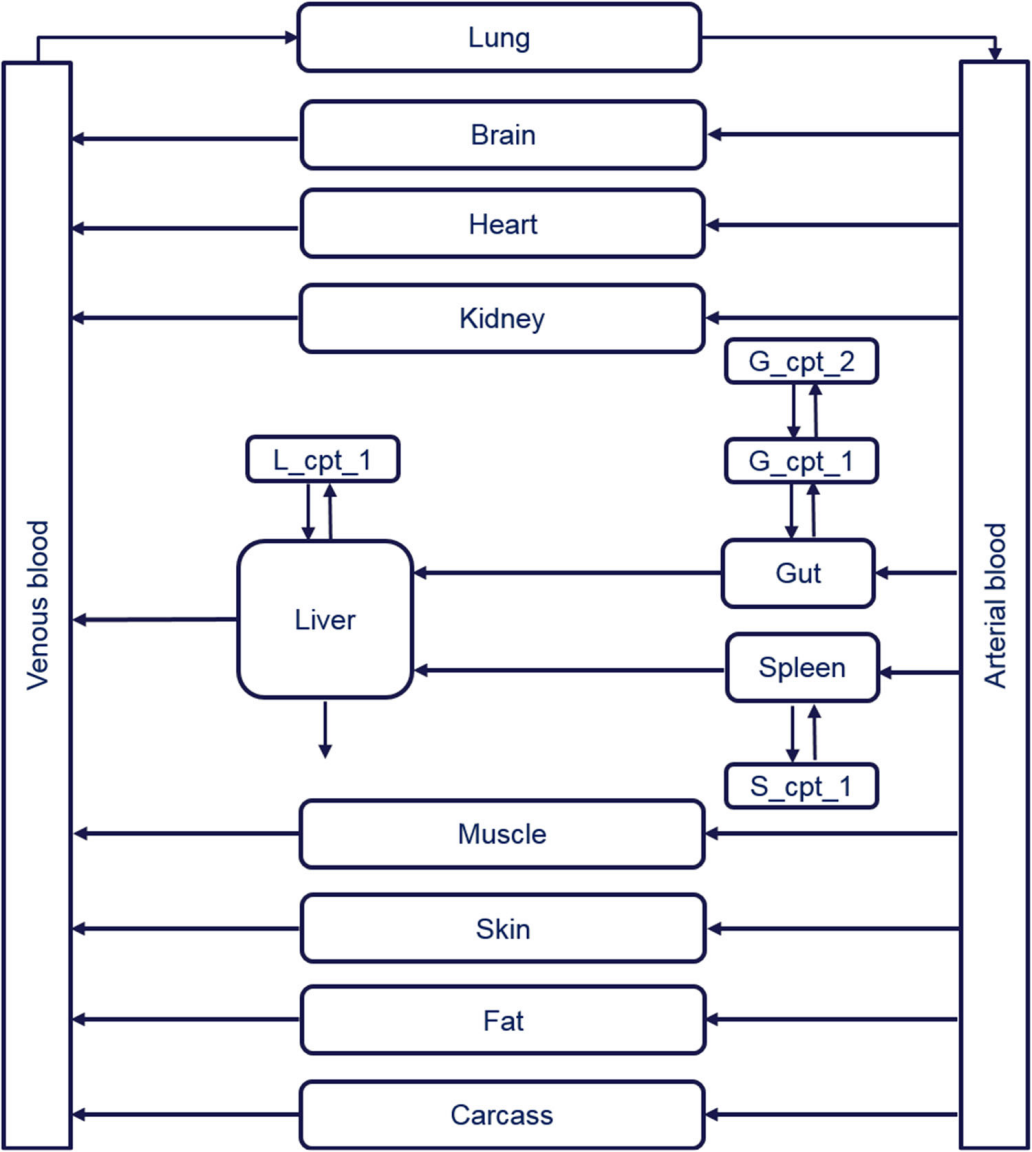
Fentanyl PBPK model structure adopted from Björkman et al. [15] (PBPK: physiologically based pharmacokinetic, L_cpt_1: an extra compartment in liver, S_cpt_1: an extra compartment in spleen, G_cpt_1: the first extra compartment in gut, G_cpt_2: the second extra compartment in gut)

A PBPK model for fentanyl was identified from the literature [15] and used as the application example. The structure of the PBPK model for fentanyl is shown in Fig. 1. This model predicted the concentrations of fentanyl in arterial blood and other tissues over time in humans after an intravenous infusion of fentanyl of 750 µg over 5 min. In the current study, the fentanyl concentrations in arterial blood were considered of importance and the concentrations in other tissues were not required.

In total there were 17 states with the liver as the site of metabolism. In addition to the usual tissues and organs that are seen in a typical PBPK model, the liver is represented by two compartments as is the spleen and the gut is represented as three compartments. These further refinements were required in the original study to improve the goodness of fit between model predictions and observed data [15]. The PBPK model contained time-invariant constants for all parameters and the model structure was linear in the parameters.

### Simulation of fentanyl PBPK model

The fentanyl PBPK model was coded as ordinary differential equations (ODEs) in MATLAB^®^ (version R2013b) based on the published model structure. Parameter values were available in the original publication [15]. The ODEs were solved using matrix exponentials to provide a closed-form for simulation.

The arterial concentration–time profile of fentanyl was simulated with an intravenous bolus of 750 µg fentanyl. Note in the original paper fentanyl was given as an arterial infusion of 750 µg over 5 min. For the simplicity of computer simulation, an arterial bolus was used. This does not affect the generality of the methods.

### Proper lumping

The proper lumping technique has previously been described by Dokoumetzidis and Aarons [16]. Briefly, a general form of the original model for a vector of model predictions (*y*) over time (*t*) is given in Eq. (1) as ODE.

1 $$\frac{dy}{dt} = K \times y$$

Here *K* is the micro-rate constant matrix in the original model of size *n *× *n* where *n* is the number of states in the original system. Parameters in the original PBPK system, recorded as blood flow, tissue volume and partitioning coefficient, were transformed to create the matrix of micro rate constants (see Sections 1A and 1B in the supplementary material 1).

In this technique the lumped micro-rate constant matrix ($\hat{K}$) can be directly obtained by the relationship of *K*, the lumping matrix (*M*) and the Moore–Penrose pseudo-inverse of lumping matrix (*M*^+^) as shown in Eq. (2).

2 $$\hat{K} = M \times K \times M^{ + }$$

The resulting lumped micro-rate constant matrix, with lumped tissues of lumped micro-rate constants that essentially average over the values from the original tissues and have the properties of the lumped perfusion, tissue volume and partitioning coefficient (see Sections 2 and 3 in the supplementary material 1), is then used to simulate the concentration–time profile in the reduced order system. The vector of ODEs for the lumped system ($\hat{y}$) are shown in Eq. (3).

3 $$\frac{{d\hat{y}}}{dt} = \hat{K} \times \hat{y}$$

The lumping matrix (*M*) transforms the states between the original and lumped systems and is a user defined *m *× *n* matrix composed of 0 s and 1 s, where *m* is the number of lumped states and *n* is the number of original states. All merged states are shown as 1 s in the same row. Here in Eq. (4) an *M* matrix for a simple example is written where the first two states of an original 3-state model are merged resulting in a 2-state lumped model.

4 $$M = \left[ {\begin{array}{*{20}c} 1 & 1 & 0 \\ 0 & 0 & 1 \\ \end{array} } \right]$$

Note the *M* matrix for the setting where the lumped model equations are the same as the original model is the identity, *I*_*n*_, of dimensions *n *× *n* (i.e. *m *= *n*).

A legal *M* matrix is specified where the sum of each column is 1 and the sum of each row ≤ *n*. In the absence of an a priori *M* matrix, an automatic search of possible *M* matrices needs to be performed to find an *M* matrix that yields a lumped model that reaches a pre-defined criterion. The optimal *M* matrix is defined as having the minimum number of states ($\hbox{min} \left( m \right); \;\;\;m \in \left\{ {1, 2, \ldots , n} \right\}$) that satisfies an acceptance criterion.

### Search algorithms for lumping matrix

#### Acceptance criterion

An acceptable lumped model was defined such that the total area under the fentanyl arterial concentration–time curve (AUC) between the lumped and original models was set to differ by 0.002% at maximum (termed ARD% for absolute value of the relative difference expressed as a percent). Note this criterion is arbitrary and any criteria can be used without loss of generality.

#### General features

Methods of searching optimal *M* matrices considered in this study include: (1) full enumeration, (2) non-adaptive random search (NARS), (3) scree plot plus NARS, and (4) simulated annealing (SA). All search algorithms were implemented in MATLAB^®^ (version R2013b).

**Fig. 2:**
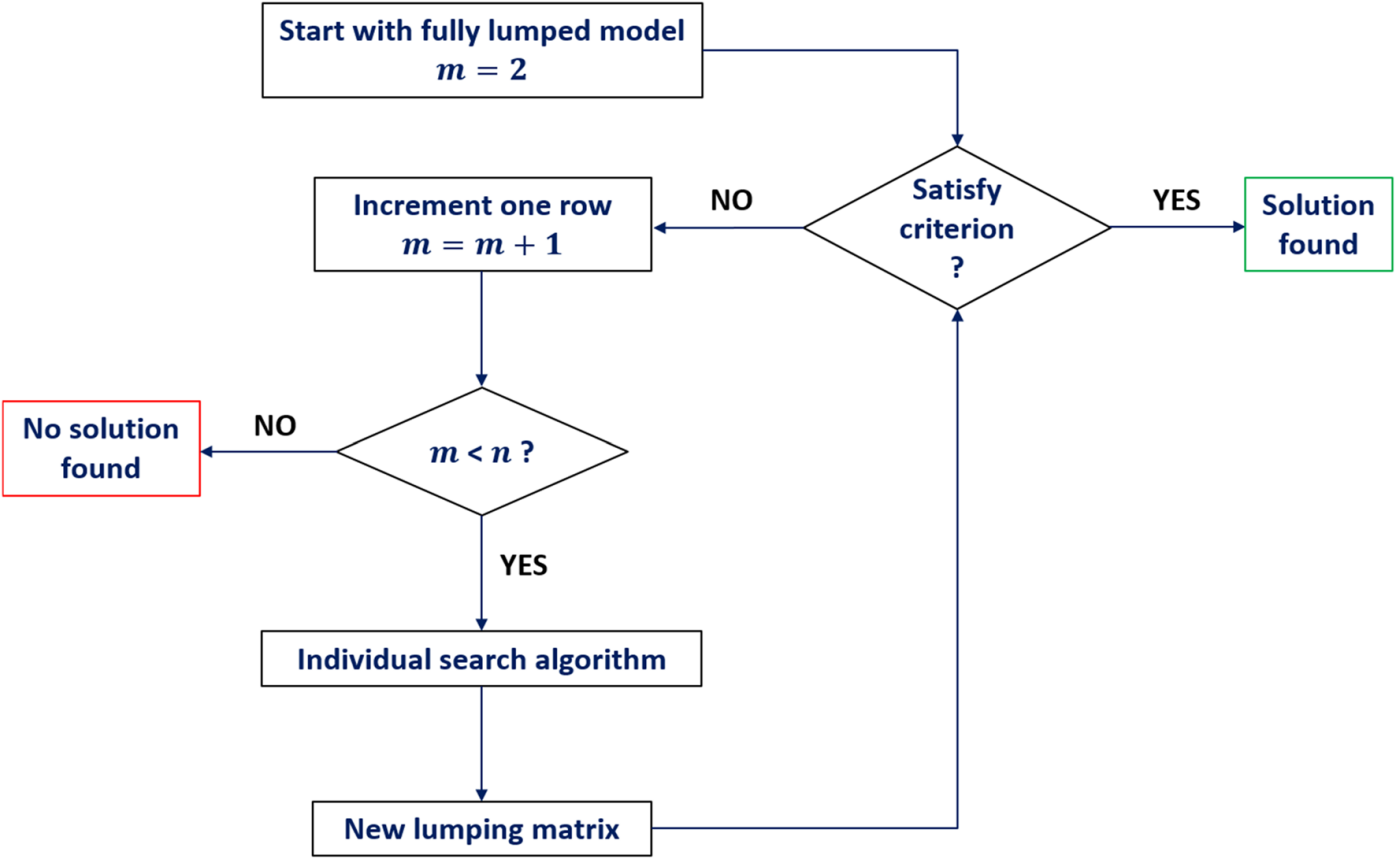
Flow chart illustrating the general features of search algorithms for a lumping matrix

In all methods arterial blood was the output state and constrained to be unlumped during the search process, as it was also the observation state, in order to illustrate a case of constrained automatic lumping. Although this choice, here, is arbitrary it is important to ensure that in future applications of automated lumping the user can control or constrain the process. All searches started from the fully lumped matrix, i.e. where states other than the output state were lumped as a single state (i.e. *m *= 2 and therefore *M* was of dimension 2 × 17). If the fully lumped state did not satisfy the acceptance criterion then the number of rows was incremented and the search algorithm was re-applied to generate new lumping matrices. Generations of new lumping matrices continued until the criterion was accepted. A flow chart is illustrated in Fig. 2.

The individual search algorithm for the best *M* matrix of size *m *× *n* was constructed by one of four techniques described below. MATLAB code (.m files) for all methods have been provided as supplementary materials.

#### Full enumeration

In full enumeration all legal lumping matrices were searched exhaustively.

Minimum ARDs% for lumped models with *m *= 3, 4, and 5 were searched respectively in full enumeration, where the total number of combinations of lumping matrices were 3^16^, 4^16^, and 5^16^ respectively (note one state (arterial blood) of the final lumped model remains unlumped).

#### Non-adaptive random search (NARS)

In NARS legal lumping matrices were constructed randomly, where random combinations of lumped states were searched.

Different numbers of random samples per row increment in *m* were evaluated, including 10, 100, 1000 or 10,000 or 100,000 or 1,000,000 samples.

#### Scree plot plus NARS

A scree plot is commonly used as a visual aid in principal component analysis to determine a number of principal components. In this visualisation the eigenvalues of the matrix of interest are plotted against the number of components of the system. This will be a monotonic curve with a downward trajectory that resembles a mountainous scree slope.

In this study a scree plot was used to visualise the influence of compartmental structures. It is constructed by plotting the rank order of the logarithm of absolute values of the eigenvalues ($log\left( {\left| {eig} \right|} \right)$) against each the relevant compartment of the *K* matrix of the original model. Either a cut-off point of $log\left( {\left| {eig} \right|} \right)$ of 0 or a change in the slope of the scree plot was used to indicate an initial estimate of the number of states in the reduced model. The resulting number of states was used for initialising NARS.

#### Simulated annealing (SA)

SA, originating from annealing metallurgy, is an optimisation method where temperature regulates the probability of acceptance of a solution (i.e. legal lumping matrices in this study). Here temperature is an artificially introduced parameter which controls the probability of moving away from the minima. More introduction to SA can be found in Goffe et al. [17].

In this study SA was used to minimise ARD% during the search of lumping matrices. Test runs were conducted to assess the conditions of the SA algorithm and to determine whether stable solutions can be found.

## Results

Four methods were considered for the search algorithm step to find the best *M* matrix for the current value of *m*. In this work full enumeration was considered the reference method as all possible solutions were considered.

### Full enumeration

A 4-state lumped model was found after 40 min where ARD% was 0.0001% satisfying the exploratory criterion (i.e. ARD% ≤ 0.002%).

The minimum ARD% for lumped models with *m *= 3 was 0.04% and the search finished within 10 min. For *m *= 4 the search of minimum ARD% (i.e. 8e−6%) took approximately 2 days. The same minimum ARD% was found for *m *= 5 while the search was completed after approximately 2 months.

### Non-adaptive random search (NARS)

The results for different numbers of random samples are summarised in Table 1. With 10 and 100 samples the random search did not find a lumped model that satisfied the criterion (i.e. the full model was the only acceptable model). With an increment of samples per iteration from 1000 to 1,000,000, the number of states in the final model that satisfied the criterion reduced. Accordingly a longer time was required for the search of larger-scale final lumped models.

**Table 1:** Number of random samples in NARS with resulting number of lumped states and time cost

Number of samples	Number of lumped states	Time cost (min)
10	–	–
100	–	–
1000	14	0.25
10,000	6	1
100,000	5	5
1,000,000	4	30

NARS non-adaptive random search

### Scree plot plus NARS

**Fig. 3:**
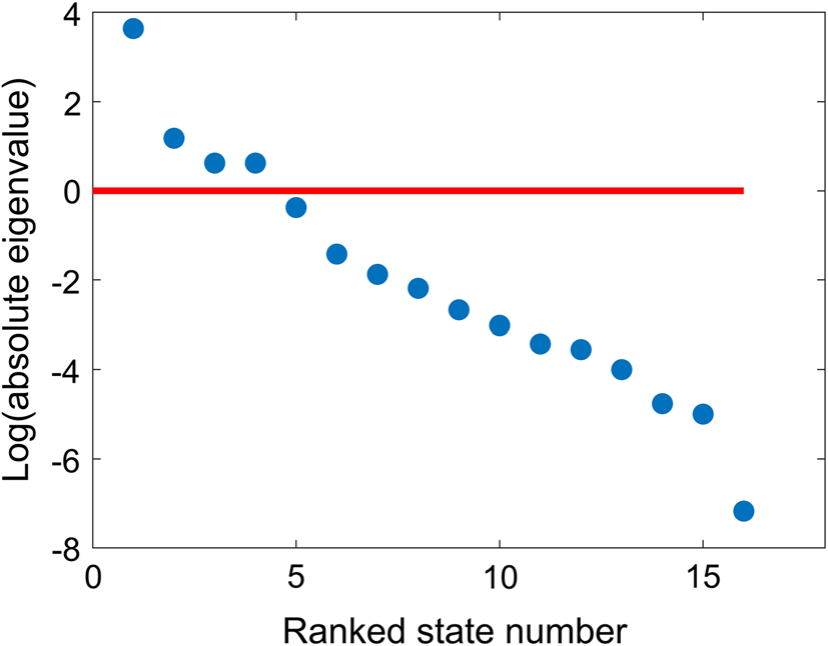
Scree plot of the original fentanyl PBPK model where logarithm of absolute values of the eigenvalues are plotted against ranked state number (horizontal line: logarithm of absolute value of eigenvalue equal to 0)

The scree plot of the original fentanyl PBPK model is shown in Fig. 3. The values of $log\left( {\left| {eig} \right|} \right)$ for the first four ranked states were above 0 and therefore may provide the basis of states that are more informative and could be left unlumped as the first iteration. The slope in the scree plot appeared to level off up to the fourth state and also indicated four or five states in the lumped model. ARD% for both lumped models were greater than 38%. The lumped states were gradually unlumped and a lumped model within the ARD% criterion was not found.

In NARS the search then started with four states (i.e. *m *= 4) with 10,000 samples, and a 7-state lumped model within the ARD% criterion was found after 40 s.

### Simulated annealing (SA)

Variables of the SA algorithm in this study were set as: (1) initial temperature = 10^4^, which regulates the probability of accepting a new energy state in the search process, (2) rate of temperature decline = 0.999 per cycle, meaning that temperature drops from 10^4^ to 10^4^ × 0.999 after the first cycle and the linear cooling scheme continues for each cycle, (3) maximum number of iterations = 2 × 10^4^ − the number of random Metropolis samples over each temperature. The stopping criteria was the same as the other methods used.

**Fig. 4:**
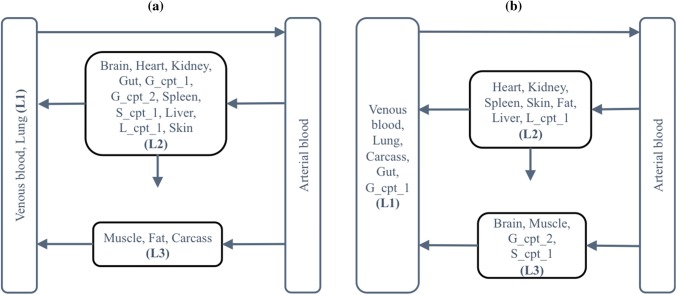
Two examples of simplified fentanyl PBPK models (PBPK: physiologically based pharmacokinetic, L_cpt_1: an extra compartment in liver, S_cpt_1: an extra compartment in spleen, G_cpt_1: the first extra compartment in gut, G_cpt_2: the second extra compartment in gut)

Five individual runs were all completed with a 4-state lumped model after approximately three minutes per run. The lumped models were not identical in structure, all with arterial blood, venous blood, and two other states resulting from different combinations of original states. Two examples of simplified fentanyl PBPK models are shown in Fig. 4. This may indicate that SA in this example was stable in returning the same number of lumped states but the form of the model varied. It should be noted that different lumped models can provide the same criterion value and hence it is possible that there is more than one lumped solution to this problem. Two additional runs with varying initial temperatures (i.e. 10^3^ and 10^5^ respectively) both gave the same outcome in terms of number of lumped states.

**Fig. 5:**
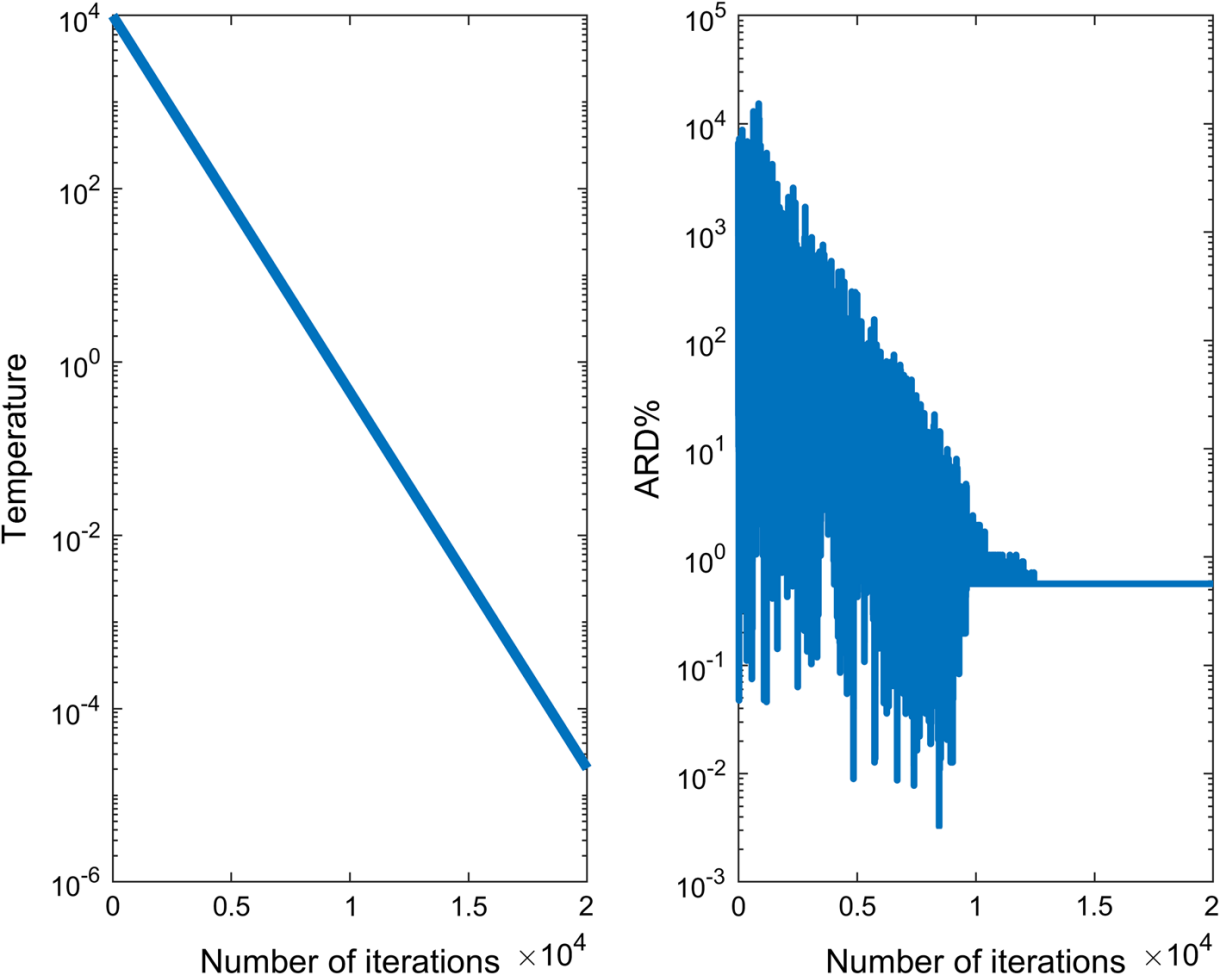
Diagnostic plots in simulated annealing algorithm (left panel: temperature change (log scale) versus iterations, right panel: ARD% (log scale) versus iterations showing convergence to optimal ARD%, ARD: absolute relative difference)

Plots in Fig. 5 show the trend of temperature change (left panel) and also the convergence to the optimal ARD% solution over iterations (right panel). With an exponential decline of temperature (i.e. annealing), a convoluted surface of ARD% over successive iterations was formed as uphill jumps were made by SA. The uphill jumps decreased as the temperature decreased and then ultimately led the search to converge to the optimal solution.

**Fig. 6:**
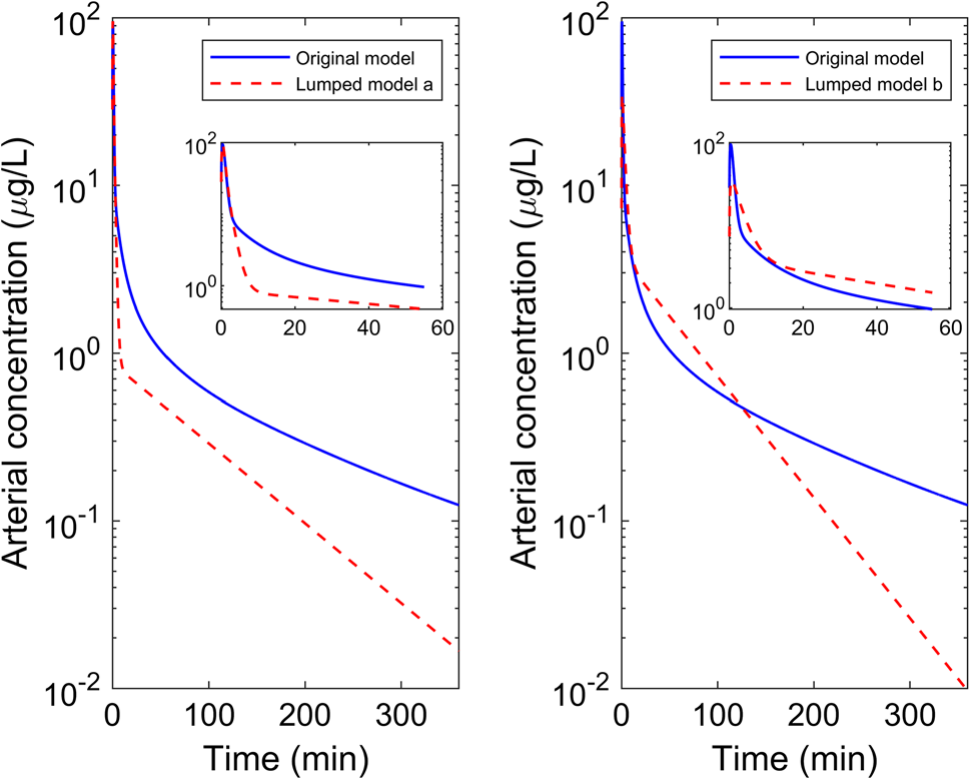
Simulated arterial concentration–time profiles for the original and lumped models (left panel: original model vs. lumped model a, right panel: original model vs. lumped model b, insert plot: concentration–time over a 60-min time scale)

**Fig. 7:**
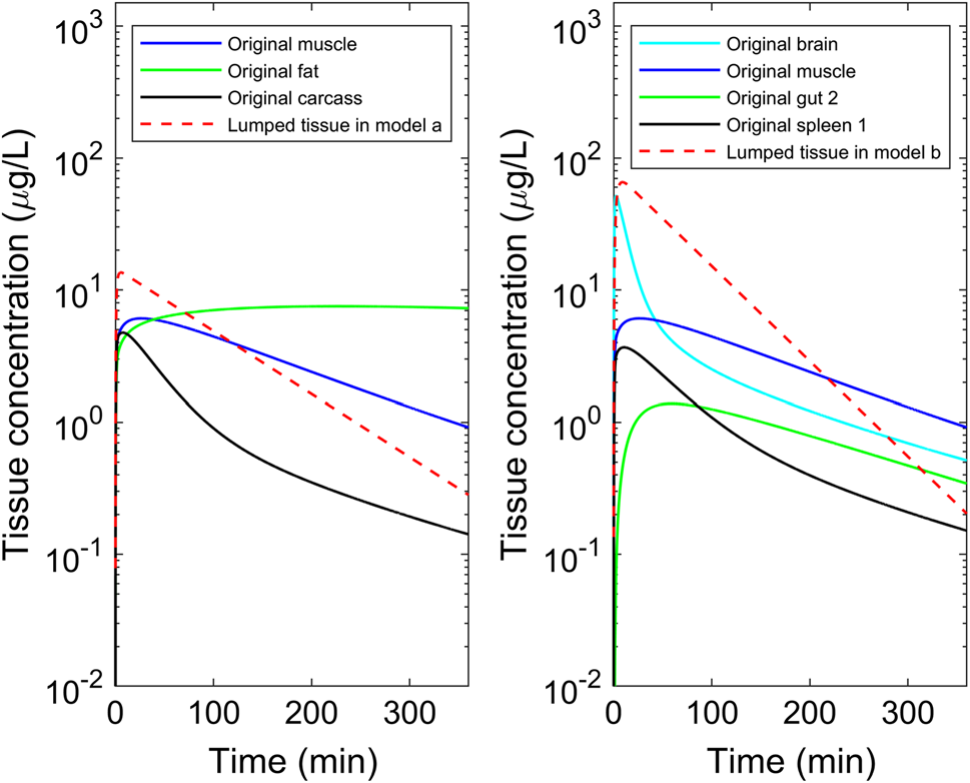
Simulated concentration–time profiles for individual tissues in the original and lumped models (left panel: original tissues vs. lumped tissue (L3) in model a, right panel: original tissues vs. lumped tissue (L3) in model b)

Plots in Fig. 6 show the simulated arterial concentration–time profiles of the two lumped models from the SA runs, in comparison to the simulated profile of the full PBPK model. Here the ARD% was set to a maximum of 0.002%. The difference in the concentration–time profile became more obvious when concentrations were lower and other criteria, e.g. sums of squares could also be considered. In Fig. 7, concentration–time profiles of individual tissues in the full PBPK model and the resulting lumped tissue were overlaid for interest. Note the lumping process was aimed to optimise for the output compartment of interest (i.e. the arterial circulation) and hence these plots represent the average lumped behaviour that was not part of the optimisation process.

## Discussion

In this study automatic proper lumping methods were explored. Various algorithms to automate the proper lumping were evaluated due to the enormous number of combinations of possible lumping matrices in the fentanyl system (i.e. in total 16^16^ = 1.8e+19), under the assumption and restriction that the output state (the state that the observations were made) was not lumped. It is possible that the output state is also lumped, in theory collapsing to a one-compartment pharmacokinetic model.

To avoid an explosion of enumeration and to improve efficiency, the search in all methods was initiated from the simplest structure (i.e. fully lumped model) and built up to more complex structures at each iteration. This search implicitly assumes that a lumped solution for the acceptance criterion of interest is readily available. However, a search starting from the full model may be more parsimonious if lumping is likely to yield an irreconcilable loss of information, i.e. most states in the finally lumped system are the same as in the original system.

Four search methods were compared, an exhaustive search, a naïve start non-adaptive random search (NARS), an informative start random search and simulated annealing (SA). The full enumeration method was set up to systematically search all combinations of lumping matrices and in effect provides a reference method. Computational time increased dramatically over the size of the problem, indicating that this method would not scale to larger problems (e.g. as *m* increased from 3 to 5 the search times increased from 10 min to 2 months). In this study, as the search started from the smallest problem and the final lumped model was of small dimension (*m *= 4), the full enumeration method was suitable. Obviously this should not be generalised to other problems. In NARS it was found that larger numbers of random samples, up to one million per iteration, converged to the optimal solution for this particular problem. However the method performed poorly with fewer samples. When NARS was paired with a scree plot to yield an informative starting point it was seen that the search completed within a short time but with a less than optimal solution. The scree plot method did not seem to confer any particular advantages in this case. In this study SA was applied to a combinatorial search across lumping matrices which represented a large-scale discrete search problem and here SA provided a stable solution, that agreed with full enumeration, within a short time.

When comparing these methods by two measures, i.e. time cost and the quality of the solution, it was found that both full enumeration and SA consistently delivered the same solution with the latter being much faster (2 months vs. 3 min). It needs to be noted that as full enumeration is a deterministic search the same lumped model was produced, while in SA different lumped models with the same number of lumped states (*m *= 4) were produced (all within the ARD% criterion but with different ARD% values). In the future, the lumped models from SA may be estimated and evaluated with respect to observed data to determine the best choice of lumped models.

Previous work has described a Bayesian method for automating a proper lumping technique with an application to a PBPK model for barbiturates [18] and a NF-κB signalling pathway model [16]. The linear PBPK model for barbiturates originally contained 19 states with most tissues assumed to be well-perfused and was initially reduced to 10 states and further down to 7 final states. The resulting lumped 7-state model, including the blood compartment for absorption and the liver compartment for elimination and the distribution into both adipose and muscle compartments, has recently been applied to describe the time course of mavoglurant plasma concentrations in a Phase-I clinical study [19]. It was evaluated in the original study that the whole PBPK model was unstable due to numerical identifiability issues when directly used to describe sparse or dense clinical data, and that fixing parameters in the whole PBPK model may produce biased estimates and underestimated uncertainty. In contrast, the lumped 7-state model retaining the physiological interpretation was found to be stable in analysing the clinical data and also in further extrapolation from adults to children.

A potential limitation to the method described in the current study is the use of ARD% which, by comparing AUCs, depends on a critical value in order that two different profiles do not generate the same AUC with different profiles. Also, and importantly, the automated search here could be used with any criterion for assessing deviation of the lumped from full model without loss of generality. We note in this work that the input is not varied (i.e. remains a single bolus dose into the arterial blood compartment) and hence ARD% for AUC while not without issues remains an effective technique for comparative purposes. One of the final lumped models (Fig. 4b) gives a reasonable arterial concentration–time profile and the lumped tissue profile has a similar profile to that of the individual tissues in the original PBPK system, although the resulting search has lost the resolution of the physiological meaning (i.e. venous blood lumped with tissues). The other lumped model (Fig. 4a) has a consistent structure with that of minimal PBPK models where venous and arterial blood compartments are separated and tissues are lumped separately [20] and the lumped tissue profile appears to capture some of the profiles of the individual tissues in the original PBPK system, the arterial concentration–time profile however displays a discrepancy at low concentrations. This is consistent with the choice of a criterion that averages over the concentration–time profile (e.g. AUC) and other criterion may be considered. The choice of a selection criterion, however, should be ultimately judged based on the intended use of the final lumped model. Since this work was not intended to provide insight into further development of fentanyl then any criterion can be used. For specific applications a criterion, e.g. sum of squares, should be considered that will support model inference and judgement. It is important, however, that the trade-off between model complexity and tolerance of the criterion be considered in the light of the use of the model. In addition, a comparison of automated proper lumping against other approaches, such as global sensitivity analysis for reducing PBPK models [5], may be considered in the future.

Nevertheless, using the model simplification techniques as in this study, large-scale linear PBPK systems may be automatically reduced to simpler structures. Since the simpler structures retain mechanistic realism they can potentially be applied to population PK studies to better predict and extrapolate the responses between species in comparison with traditional empirical approaches [2]. Additionally, simpler model structures may be used to optimise the design of PK experiments while the original PBPK models may be limited by their structural complexity requiring intensive sampling designs for estimating all parameters [21, 22].

## Conclusion

In this study automated proper lumping methods were used to simplify an existing linear fentanyl PBPK model, and simulated annealing was found to a robust and efficient of the algorithm. This approach could be considered for the simplificaiton of other types of PBPK models.

## Electronic supplementary material

Below is the link to the electronic supplementary material.

Supplementary material 1 (DOCX 44 kb)

Supplementary material 2 (PDF 64 kb)

Supplementary material 3 (m 1 kb)

Supplementary material 4 (m 2 kb)

Supplementary material 5 (m 2 kb)

Supplementary material 6 (m 1 kb)

Supplementary material 7 (m 1 kb)

Supplementary material 8 (m 11 kb)

Supplementary material 9 (m 3 kb)

Supplementary material 10 (m 2 kb)

Supplementary material 11 (m 2 kb)

Supplementary material 12 (m 2 kb)

Supplementary material 13 (m 1 kb)

Supplementary material 14 (m 3 kb)

Supplementary material 15 (m 1 kb)

Supplementary material 16 (m 2 kb)

Supplementary material 17 (m 2 kb)

Supplementary material 18 (m 11 kb)

Supplementary material 19 (m 1 kb)

Supplementary material 20 (m 1 kb)

Supplementary material 21 (m 2 kb)

Supplementary material 22 (m 2 kb)
