# Supplementary material for: Automated proper lumping for simplification of linear physiologically based pharmacokinetic systems
Source: J Pharmacokinet Pharmacodyn. 2019 Jun 21;46(4):361–70. doi: 10.1007/s10928-019-09644-5 (PMC6656793; doi:10.1007/s10928-019-09644-5)
Supplement: Supplementary file 1 — Supplementary material 1 (DOCX 44 kb) [file 10928_2019_9644_MOESM1_ESM.docx]

Supplementary materials

Title: Automated proper lumping for simplification of linear physiologically based pharmacokinetic systems

Journal name: Journal of Pharmacokinetics and Pharmacodynamics

**Authors:** Shan Pan^1,2^, Stephen B. Duffull^1^

^1^School of Pharmacy, University of Otago, Dunedin, New Zealand

^2^St John’s Institute of Dermatology, Guy’s and St Thomas’ NHS Foundation Trust, London, United Kingdom

**Corresponding author:**

Shan Pan

Guy’s and St Thomas’ NHS Foundation Trust

Great Maze Pond

London

SE1 7EH

United Kingdom

Email address: [shan.pan@kcl.ac.uk](mailto:shan.pan@kcl.ac.uk)

Section 1: Parameter values from the original fentanyl PBPK model

*1A: Parameterisation of the original PBPK system*

*1B: Derived matrix of micro-rate constants*

Section 2: Parameter values from the lumped fentanyl PBPK model (model structure in Figure 4a)

Section 3: Parameter values from the lumped fentanyl PBPK model (model structure in Figure 4b)

Section 4: MATLAB^®^ code of the four methods for automatic model simplification

Section 1: Parameter values from the original fentanyl PBPK model

*1A: Parameterisation of the original PBPK system*

% Adopted from Bjorkman et al. 1994

WT = 73; % average weight for man, unit: kg

Qcad = 6.7; % cardiac output, unit: L/min

Vven = 3.9; % volume/weight of vein, unit: kg

Vart = 2.0; % volume/weight of artery, unit: kg

Vlun = 0.47; % volume/weight of lung, unit: kg

Qlun = 14.3*Vlun; % blood flow in lung, unit: L/min

KPlun = 15.3; % partition coeffient for brain, unit: ml/g

KTlun = Qlun/(Vlun*KPlun);

Vbra = 1.4; % volume/weight of brain, unit: kg

Qbra = 0.56*Vbra; % blood flow in brain, unit: L/min

KPbra = 4; % partition coeffient for brain, unit: ml/g

KTbra = Qbra/(Vbra*KPbra);

Vhea = 0.33; % volume/weight of heart, unit: kg

Qhea = 0.8*Vhea; % blood flow in heart, unit: L/min

KPhea = 5.1; % partition coeffient for heart, unit: ml/g

KThea = Qhea/(Vhea*KPhea);

Vkid = 0.31; % volume/weight of kidney, unit: kg

Qkid = 4*Vkid; % blood flow in kidney, unit: L/min

KPkid = 13.7; % partition coeffient for kidney, unit: ml/g

KTkid = Qkid/(Vkid*KPkid);

Vliv = 1.8; % volume/weight of liver, unit: kg

Qliv = 0.96*Vliv; % blood flow in liver, unit: L/min

KPliv = 4.3; % partition coeffient for liver, unit: ml/g

KTliv = Qliv/(Vliv*KPliv);

Vmus = 30; % volume/weight of muscle, unit: kg

Qmus = 0.038*Vmus; % blood flow in muscle, unit: L/min

KPmus = 3.5; % partition coeffient for muscle, unit: ml/g

KTmus = Qmus/(Vmus*KPmus);

Vski = 3.3; % volume/weight of skin, unit: kg

Qski = 0.12*Vski; % blood flow in skin, unit: L/min

KPski = 2.3; % partition coeffient for skin, unit: ml/g

KTski = Qski/(Vski*KPski);

Vfat = 12.5; % volume/weight of fat, unit: kg

Qfat = 0.028*Vfat; % blood flow in fat, unit: L/min

KPfat = 30.3; % partition coeffient for fat, unit: ml/g

KTfat = Qfat/(Vfat*KPfat);

Vcar = 15.5; % volume/weight of carcass, unit: kg

Qcar = 0.04*Vcar; % blood flow in carcass, unit: L/min

KPcar = 1; % (not avaialbe) partition coeffient for carcass, unit: ml/g

KTcar = Qcar/(Vcar*KPcar);

Vgut = 1.2; % volume/weight of gut, unit: kg

Vsint = Vgut; % volume/weight of samll intestine, unit: kg

Qgut = 0.83*Vgut; % blood flow in gut, unit: L/min

Qsint = Qgut; % blood flow in small intestine, unit: L/min

KPgut = 9; % partition coeffient for gut, unit: ml/g

KTgut = Qgut/(Vgut*KPgut);

Vpas = (0.18+0.10)/2; % average volume/weight of pancreas and spleen, unit: kg

Qpas = (0.6+1.2)/2*Vpas; % average blood flow of pancreas and spleen, unit: L/min

KPpas = (24.1+31.3)/2; % average partition coeffient of pancreas & spleen, unit: ml/g

KTpas = Qpas/(Vpas*KPpas);

CL12_pas = 2.21*Vpas;% clearance in pancrea + spleen model, unit: L/min (in paper unit was 2.21 ml/min per g)

Vpas_1 = 0.082*Vpas; % see page 388, volume of 1st cpt in pancreas + spleen model, unit: L (in paper unit was 0.082 ml per g)

Vpas_2 = 22.3*Vpas; % volume of 2nd cpt in pancreas + spleen model, unit: L (in paper unit was 22.3 ml per g)

CL12_hep = 0.35*Vliv; % clearance in liver model, unit: L/min (in paper unit was 0.35 ml/min per g)

CL10_hep = 0.095*Vliv; % clearance in liver model, unit: L/min (in paper unit was 95 ml/min per kg)

Vhep_2 = 8.7*Vliv; % volume of 2nd cpt in liver model, unit: L (in paper unit was 8.7 ml per g)

CL12_gut = 0.89*Vgut; % clearance in gut model, unit: L/min (in paper unit was 0.89 ml/min per g)

CL21_gut = CL12_gut;

CL21_hep = CL12_hep;

CL21_pas = CL12_pas;

CL23_gut = 0.03*Vgut;% clearance in gut model, unit: L/min (in paper unit was 0.03 ml/min per g)

CL32_gut = CL23_gut;

Vgut_1 = Vgut;

Vgut_2 = 6.4*Vgut; % volume of 2nd cpt in gut model, unit: L (in paper unit was 6.4 ml per g)

Vgut_3 = 3.26*Vgut; % volume of 3rd cpt in gut model, unit: L (in paper unit was 3.26 ml per g)

*1B: Derived matrix of micro-rate constants*

K=

[-Qcad/Vven 0 KTbra*Vbra/Vven KThea*Vhea/Vven KTkid*Vkid/Vven KTliv*Vliv/Vven KTmus*Vmus/Vven KTski*Vski/Vven ...

KTfat*Vfat/Vven KTcar*Vcar/Vven 0 0 0 0 0 0;

Qcad/Vart -Qcad/Vart 0 0 0 0 0 0 ...

0 0 0 0 0 0 0 0;

0 Qbra/Vbra -KTbra 0 0 0 0 0 .. 0 0 0 0 0 0 0 0;

0 Qhea/Vhea 0 -KThea 0 0 0 0 ...

0 0 0 0 0 0 0 0;

0 Qkid/Vkid 0 0 -KTkid 0 0 0 ...

0 0 0 0 0 0 0 0;

0 0 0 0 0 Loss_liver 0 0 ...

0 0 S11_to_Liver 0 0 S14_to_Liver 0 S16_to_Liver;

0 Qmus/Vmus 0 0 0 0 -KTmus 0 ...

0 0 0 0 0 0 0 0;

0 Qski/Vski 0 0 0 0 0 -KTski ...

0 0 0 0 0 0 0 0;

0 Qfat/Vfat 0 0 0 0 0 0 ...

-KTfat 0 0 0 0 0 0 0;

0 Qcar/Vcar 0 0 0 0 0 0 ...

0 -KTcar 0 0 0 0 0 0;

0 Qsint/Vgut_1 0 0 0 0 0 0 ...

0 0 Loss_gut_1 CL21_gut/Vgut_1 0 0 0 0;

0 0 0 0 0 0 0 0 ...

0 0 CL12_gut/Vgut_2 Loss_gut_2 CL32_gut/Vgut_2 0 0 0;

0 0 0 0 0 0 0 0 ...

0 0 0 CL23_gut/Vgut_3 -CL32_gut/Vgut_3 0 0 0;

0 Qpas/Vpas_1 0 0 0 0 0 0 ...

0 0 0 0 0 Loss_pas_1 CL21_pas/Vpas_1 0;

0 0 0 0 0 0 0 0 ...

0 0 0 0 0 CL12_pas/Vpas_2 -CL21_pas/Vpas_2 0;

0 0 0 0 0 CL12_hep/Vhep_2 0 0 ...

0 0 0 0 0 0 0 -CL21_hep/Vhep_2];

Section 2: Parameter values from the lumped fentanyl PBPK model (model structure in Figure 4a)

% Matrix of micro rate constants in the lumped model

% Lumped matrix of micro rate constants were produced from the original matrix of micro rate constants together with lumping matrix

% Original matrix of micro rate constants were derived from parameters in the original PBPK system that was recorded as blood flow, tissue volume and partitioning coefficient (see sections 1A and 1B)

% Lumped micro-rate constants in lumped tissues essentially average over the values from the original tissues and have the properties of the lumped perfusion, tissue volume and partitioning coefficient

K = [-1.72 0 0.02 0.08

3.35 -3.35 0 0

0 17.3 -1.15 0

0 0.11 0 -0.02]

% volume of artery remains the same (unlumped)

Vart = 2.0; % volume/weight of artery, unit: kg

Section 3: Parameter values from the lumped fentanyl PBPK model (model structure in Figure 4b)

% Matrix of micro rate constants in the lumped model

% Lumped matrix of micro rate constants were produced from the original matrix of micro rate constants together with lumping matrix

% Original matrix of micro rate constants were derived from parameters in the original PBPK system that was recorded as blood flow, tissue volume and partitioning coefficient (see sections 1A and 1B)

% Lumped micro-rate constants in lumped tissues essentially average over the values from the original tissues and have the properties of the lumped perfusion, tissue volume and partitioning coefficient

K = [-0.61 0.87 0.03 0.03

0.84 -3.35 0 0

0.002 0.60 -0.06 0.01

0.02 15.9 6.74 -5.53]

% volume of artery remains the same (unlumped)

Vart = 2.0; % volume/weight of artery, unit: kg

Section 4: MATLAB^®^ code of the four methods for automatic model simplification

### Main files

*Runfile_Autolumping_FenPBPK.m*

% Initial condition for each cpt (vector)

Dose = 750; %microgram

IC = [Dose/Vven; zeros(15,1)]; % unit: ug/L

% Indicate the input state

%input = [];

% Indicate the output state (e.g. output = [2] meant the 2nd state for observation)

output = [2];

% Volume of output state

V = [1]; % unit:L

% Time range of interest

TT = [0:0.5:210]; % unit:min

% Indicate the criterion for accepting the lumped model

% criterion_option: 1 for absolute relative difference in AUC over TT, 2 for absolute relative difference in observed conc-time curve

% criterion_limit: the upper limit in relatice difference, e.g.criterion_limit = [20] means 20%

criterion_option = [1];

criterion_limit = [0.002];

crit_unlumped %calculate AUC of original unlumped system

Model_parameter_values %parameter values in original paper

%% Report the following outputs to users

% report vector and ARD%

[ARD, Vbest] = Output_function(K,IC,output,TT,criterion_option,criterion_limit);

disp(Vbest)

disp(strcat('ARD=',num2str(ARD),'%'))

*OBJV_function.m*

function [ ARD,A_hat,output_row ] = OBJV_function(M,K,IC,TT,output,criterion_option,criterion_limit,AUC)

K_hat = M*K*pinv(M);

IC_hat = M*IC;

A_hat = ME_solution_function(K_hat,IC_hat,TT);

% choose the amt in output state over all time points in TT for the calculation of OBJV

% first need to find which row the output state is in now

output_row = find(M(:,output)==1);

if criterion_option ==1 % absolute relative difference (%) in AUC

AUC_hat = cumtrapz(TT,A_hat(output_row,:));

AUC_hat = AUC_hat(length(AUC_hat));

ARD = abs((AUC-AUC_hat)/AUC)*100;

else

ARD = max(abs((A(output,:)-A_hat(output_row,:))./A(output,:))*100);

end

end

*ME_solution_function.m*

function A = ME_solution(K,IC,TT)

% for liner ODEs: dy/dt=K*y --> transformed into matrix exponential: dy/dt=exp(t*K)*y(0)

% matrix exponential solution requires eigenvalues and eigenvectors of rate constant matrix

[P_m,Lam_m]=eig(K);

% eigenvalue/eigenvector decomposition for each state individually

q_m=inv(P_m)*IC;

R_m = zeros(length(q_m),length(TT));

for j_m=1:length(q_m)

R_m(j_m,:)=exp(Lam_m(j_m,j_m)*TT)*q_m(j_m);

end

% obtain matrix of amounts in all cpts: row for cpt number, column for time point

A = P_m*R_m;

*matrix2vector.m*

function [ V ] = matrix2vector( M )

[m,n] = size(M);

V=zeros(1,n);

for i=1:n

for j=1:m

if (M(j,i)==1)

V(i) = j;

break;

end

end

end

end

*vector2matrix.m*

function [ M ] = vector2matrix( V, size)

M = zeros(size, length(V));

for (j=1:length(V))

M(V(j),j) = 1;

end

end

*crit_unlumped.m*

A = ME_solution_function(K,IC,TT);

AUCc = cumtrapz(TT,A(output,:));

AUC = AUCc(length(AUCc));

### Full enumeration

*Autolumping_Matrix_function.m*

function [M,ARD,simulation] = Autolumping_Matrix_function(K,IC,output,TT,criterion_option,criterion_limit)

% This function is to update the M matrix for autolumping

% Number of columns in the original K matrix

[~,Column] = size(K);

% % Initialisation

% OBJV_best = 1000000;

% ARD = 1000000; % ARD: absolute relative difference

% starting randomly generate the permulation for each randperm within the maxcount

for n = 1:Column-1 % n is the number of rows

if n ==1 % fully lumped model -- then compare OBJV

M = ones(1,Column);

[ ARD,A,A_hat,output_row ] = OBJV_function(M,K,IC,TT,output,criterion_option,criterion_limit);

% if absolute relative difference satisfies the criterion then stop

if ARD <= criterion_limit

break

end

else

% enumerate all lumping matrix for each number of row using findLumpingMatrix_function

Column_new = Column-1;

[ARD,M,A,A_hat,output_row] = findLumpingMatrix_function ([], [1:n], Column_new,K,IC,TT,output,criterion_option,ARD, criterion_limit);

% calculations of OBJV & absolute relative difference are inside the function of findLumpingMatrix

end

% if satisfies the criterion then break

if ARD <= criterion_limit

break

end

end

% output from this function -- output conc over TT for original and final lumped model

simulation(1,:) = A(output,:);

simulation(2,:) = A_hat(output_row,:);

end

*findLumpingMatrix_function.m*

function[ARD,M,A,A_hat,output_row] = findLumpingMatrix_function (P, S, Column_new,K,IC,TT,output,criterion_option,ARD, criterion_limit)

%P: state of the permutation, S: elements we can choose from

if Column_new == 0 % a legal lumping matrix is found

P

row = max(S);

M = zeros(row,length(P));

for i = 1:length(P)

M(P(i),i)=1;

end

% add the constrained output state into lumping matrix

add_output_col = zeros(row,1);

add_output_row = zeros(1,length(P)+1);

add_output_row(1,output) = 1;

M = [M(:,1:output-1), add_output_col , M(:,output:length(P))]; % update with a new column of zeros

M = [M ; add_output_row]; % update with another row for constraining the output state

% calculate OBJV for M

[ ARD,A,A_hat,output_row ] = OBJV_function(M,K,IC,TT,output,criterion_option,criterion_limit);

% if absolute relative difference satisfies the criterion then stop

if ARD <= criterion_limit

return

end

else

for i = 1: length(S)

P0 = [P, S(i)];

[ARD,M,A,A_hat,output_row] =findLumpingMatrix_function (P0, S, Column_new-1,K,IC,TT,output,criterion_option,ARD, criterion_limit); % repeat the function

if ARD <= criterion_limit

return

end

end

end

end

### Non-adaptive random search (NARS)

*Autolumping_Matrix_function.m*

function [M,ARD,simulation] = Autolumping_Matrix_function(K,IC,output,TT,criterion_option,criterion_limit)

% This function is to update the M matrix for autolumping (see details in the flowchart)

% Number of columns in the original K matrix

[~,Column] = size(K);

% Initialisation

% OBJV_best = 1000000;

% ARD = 1000000;

% translate M matrix into randPerm, e.g. for M = [1,0,0;0,1,1], it is equivalent to randPerm = [1,2,2] (1st 1 at row 1, 2nd and 3rd 1s at row 2)

randPerm = zeros(1,Column);

% starting randomly generate the permulation for each randperm within the maxcount

for m = 1:Column-1 % n is the number of rows

m

count = 0;

if m ==1 % if only 1 row, meaning fully lumped model -- then compare OBJV

M = ones(1,Column);

[ ARD,A,A_hat,output_row ] = OBJV_function(M,K,IC,TT,output,criterion_option,criterion_limit);

% if satisfies the criterion then stop

if ARD <= criterion_limit

break

end

else

% Maximum counts to avoid endless loop

maxcount = 10^6; % maximum number of random sampling

randPerm_collection=ones(1,Column-1); % collect the randperm

while count <= maxcount

M = zeros(m,Column-1);% translate randperm back into M matrix

for i = 1:Column-1 % randomly construct leagal candidateMatrices

randPerm(i) = randi([1,m]); % randomly assign a value to each element in randPerm

end

% if ismember(randPerm,randPerm_collection,'rows')~=1 % if the generated randPerm is not in the collection then collect it

% randPerm_collection = [randPerm_collection;randPerm];

% translate randPerm back into M matrix -- fill the ith column && row of randperm(i) with "1"

for i = 1:Column-1

M(randPerm(i),i)=1;

end

% add the constrained output state into lumping matrix

add_output_col = zeros(m,1);

add_output_row = zeros(1,Column);

add_output_row(1,output) = 1;

M = [M(:,1:output-1), add_output_col , M(:,output:Column-1)]; % update with a new column of zeros

M = [M ; add_output_row]; % update with another row for constraining the output state

% calculate OBJV for the randPerm that is in the collection -- which means do not repeat OBJV calculation for the repeated randPerm

[ ARD,A,A_hat,output_row ] = OBJV_function(M,K,IC,TT,output,criterion_option,criterion_limit);

% if satisfies the criterion then stop

if ARD <= criterion_limit

break

end

count = count + 1;

end

end

% if satisfies the criterion then break

if ARD <= criterion_limit

break

end

end

% output from this function -- output conc over TT for original and final lumped model

simulation(1,:) = A(output,:);

simulation(2,:) = A_hat(output_row,:);

end

### Screeplot

*Fentanyl_PBPK_screeplot.m*

abs_eig = -eig(K);

rank_abs_eig = sort(abs_eig,'descend');

% ranked states: 1, 2, 3, 4, 5, 6, 7, 8, 9, 10, 11, 12, 14, 16, 15, 13

### Simulated annealing (SA)

*Autolumping_Matrix_function.m*

function [Vbest,ARD,simulation] = Autolumping_Matrix_function(K,IC,output,TT,criterion_option,criterion_limit,AUC)

% initialisation for lumping matrix

M=[ 0 1 0 0 0 0 0 0 0 0 0 0 0 0 0 0

1 0 1 1 1 1 1 1 1 1 1 1 1 1 1 1

0 0 0 0 0 0 0 0 0 0 0 0 0 0 0 0];

[ARD,A_hat,output_row ] = OBJV_function(M,K,IC,TT,output,criterion_option,criterion_limit,AUC);

while ARD > criterion_limit

[r c]=size(M);

V = matrix2vector(M);

nrows = r;

Vbest=V;

M=vector2matrix(V, nrows);

[ARD,A_hat,output_row ] = OBJV_function(M,K,IC,TT,output,criterion_option,criterion_limit,AUC);

ARDbest = ARD;

constraint = [2,1]; %constraint on output state: first row, second column

% SA initialisation

maxit = 5000*4;

itis = 1;

temp = 10000; % 1000, 10000, 100000 %temperature change here

decayRate =0.999;

repeat = 10; % for repeated heating before next annealing step

% For plotting

ARDbestHist = [];

ARDHist = [];

tempHist = [];

%% SA

while itis < maxit

for i = 1:repeat

VNew = nearestNeighbour(V, nrows, constraint);

MNew = vector2matrix(VNew,nrows);

[ARDnew,A_hat,output_row] = OBJV_function(MNew,K,IC,TT,output,criterion_option,criterion_limit,AUC);

p = exp(-(ARDnew-ARD)/temp);% Boltzman factor

if p > rand

V = VNew;

ARD = ARDnew;

end

if ARD < ARDbest

Vbest = V;

ARDbest = ARD;

A_hat_best = A_hat; % keep record of simulation for best model

end

end

temp=temp*decayRate;

ARDbestHist(itis) = ARDbest;

ARDHist(itis) = ARD;

tempHist(itis) = temp;

itis = itis+1;

if rem(itis,5000)==0

r

itis

end

end

M = [M;zeros(1,c)]; % a new M with additonal row of zeros

ARD=ARDbest;

end

crit_unlumped %calculate AUC of original unlumped system

simulation(1,:) = A(output,:);

simulation(2,:) = (A_hat_best(output_row,:));

end

*nearestNeighbour.m*

function [ Mnew ] = nearestNeighbour( M , rows , constraint)

Mnew = M;

[x,cols] = size(M);

i = randsample([1:constraint(1)-1, constraint(1)+1:cols],1);

j = randsample([1:constraint(2)-1, constraint(2)+1:rows],1);

while M(i) == j

j = randsample([1:constraint(2)-1, constraint(2)+1:rows],1);

end

Mnew(i) = j;

end
