# Supplementary material for: Automated proper lumping for simplification of linear physiologically based pharmacokinetic systems
Source: J Pharmacokinet Pharmacodyn. 2019 Jun 21;46(4):361–70. doi: 10.1007/s10928-019-09644-5 (PMC6656793; doi:10.1007/s10928-019-09644-5)
Supplement: Supplementary file 2 — Supplementary material 2 (PDF 64 kb) [file 10928_2019_9644_MOESM2_ESM.pdf]

************************************ READ ME ************************************

Title: Automated proper lumping for simplification of linear physiologically based pharmacokinetic systems

Journal name: Journal of Pharmacokinetics and Pharmacodynamics

Authors: Shan Pan[1,2], Stephen B. Duffull[1]
[1]School of Pharmacy, University of Otago, Dunedin, New Zealand
[2]St John's Institute of Dermatology, Guy's and St Thomas' NHS Foundation Trust, London, United Kingdom

Corresponding author:
Shan Pan
Guy's and St Thomas' NHS Foundation Trust
Great Maze Pond
London
SE1 7EH
United Kingdom
Email address: shan.pan@kcl.ac.uk

************************************ READ ME ************************************

** Created by: Shan Pan

** Date: 27-May-2019

** Content purpose: to explain what .m files to run for the individual methods

- ***Full_Enumeration files***

1. FE_Runfile_Autolumping_FenPBPK.m : this is the run file to define the inputs
2. Model_parameter_values.m : this is the script to define parameters and parameter values
3. FE_Autolumping_Mmatrix_function.m : this is the function file for initialising lumping matrix enumeration
4. findLumpingMatrix_function.m : this is the function file to enumerate all solutions of lumping matrix
5. FE_OBJV_function.m : this is the function file to define the criterion of accepting a lumped model
6. ME_solution_function.m : this is the function file for matrix exponential solution
7. FE_Output_function.m : this is the function file to define the outputs to be delivered

- ***NARS_Plus_Screeplot files***

1. NARS_Runfile_Autolumping_FenPBPK.m : this is the run file to define the inputs
2. Model_parameter_values.m : this is the script to define parameters and parameter values
3. NARS_Autolumping_Mmatrix_function.m : this is the function file for initialising lumping matrix enumeration
4. NARS_OBJV_function.m : this is the function file to define the criterion of accepting a lumped model
5. ME_solution_function.m : this is the function file for matrix exponential solution
6. NARS_Output_function.m : this is the function file to define the outputs to

be delivered

7. Screeplot_Fentanyl_PBPK.m : this is the run file to generate screeplot

- ***Simulated_Annealing files***

1. SA_Runfile_Autolumping_FenPBPK.m : this is the run file to define the inputs
2. Model_parameter_values.m : this is the script to define parameters and parameter values
3. SA_Autolumping_Mmatrix_function.m : this is the function file for initialising lumping matrix enumeration
4. SA_OBJV_function.m : this is the function file to define the criterion of accepting a lumped model
5. ME_solution_function.m : this is the function file for matrix exponential solution
6. SA_Output_function.m : this is the function file to define the outputs to be delivered
7. crit_unlumped.m : this is the function file to calculate AUC of the original unlumped system
8. matrix2vector.m : this is the function file to convert matrix into vector
9. nearestNeighbour.m : this is the function file to find the nearest neighbour solution
10. vector2matrix.m : this is the function file to convert from vector to matrix
